# Comparative evaluation of published real-time PCR assays for the detection of malaria following MIQE guidelines

**DOI:** 10.1186/1475-2875-12-277

**Published:** 2013-08-08

**Authors:** Saba Alemayehu, Karla C Feghali, Jessica Cowden, Jack Komisar, Christian F Ockenhouse, Edwin Kamau

**Affiliations:** 1 Military Malaria Research Program, Malaria Vaccine Branch, Walter Reed Army Institute of Research, 503 Robert Grant Ave, Silver Spring, MD, USA

## Abstract

**Background:**

The use of malaria-specific quantitative real-time PCR (qPCR) is increasing due to its high sensitivity, speciation and quantification of malaria parasites. However, due to the lack of consensus or standardized methods in performing qPCR, it is difficult to evaluate and/or compare the quality of work reported by different authors for a cross-study and/or cross-platform assay analysis.

**Methods:**

The performances of seven published qPCR assays that detect *Plasmodium spp* or *Plasmodium falciparum* were compared using standard DNA and samples from a clinical trial. Amplification and qPCR measurements were performed using the Applied Biosystems 7500 Fast Real-Time PCR System. All the analyses were automatically established using the default settings. For the TaqMan probe format, the assays were performed in the background of QuantiFast Probe Master Mix whereas in SYBR Green format, the assays were performed in the background of QuantiFast SYBR Green Master Mix and QuantiTect SYBR Green Master Mix background.

**Results:**

Assays with high PCR efficiencies outperformed those with low efficiencies in all categories including sensitivity, precision and consistency regardless of the assay format and background. With the exception of one assay, all assays evaluated showed lower sensitivity compared to what have been published. When samples from a malaria challenge study were analysed, the qPCR assay with the overall best performance detected parasites in subjects earliest and with most consistency.

**Conclusion:**

The data demonstrate the need for increased consensus and guidelines that will encourage better experimental practices, allowing more consistent and unambiguous interpretation of qPCR results.

## Background

The gold standard method for malaria diagnosis is microscopy [1,2]. However, numerous challenges associated with performing quality microscopy may lead to variations in assay sensitivity and specificity [3,4] affecting patient diagnostic outcome and/or clinical trial results [5]. Microscopy is highly operator-dependent and proficiency testing is required to achieve reproducible, high-quality data [6]. Molecular assays that detect *Plasmodium*-specific nucleic acid sequences are increasingly being implemented in order to overcome some of the limitations associated with microscopy. These assays are several orders of magnitude more sensitive than microscopy or antigen detection tests [7,8]. Malaria-specific applications of quantitative real-time PCR (qPCR) have allowed for identification, speciation and quantification of malaria parasites [7-14]. Notably, qPCR is increasingly being used to analyse pre-patent parasitaemia in controlled human malaria infection (CHMI) trials as well as evaluation of low parasitaemia in field studies [15-19]. PCR can also be used as a tool for identification of asymptomatic carriers [20].

Most of the qPCR assays that have been described for detection of *Plasmodium* target the multicopy 18S ribosomal RNA (rRNA) genes [21]. Other targets such as mitochondrial genes, *var* and *stevor* genes have also been described [13,21]. These assays are designed as monoplex where they amplify a single target or as multiplex, where they amplify two targets or more. When used as a monoplex assay, the reported detection limit ranges from about 0.002 to 30 parasites/μL [14,21] whereas as a multiplex, the detection limit ranges from 0.2 to 5 parasites/μL [7,8,14,21]. Hermsen *et al*. [9] and Lee *et al.*[10] described some of the early monoplex qPCR assays for detection of *Plasmodium spp* with a detection limit of 0.02 parasites/μL and 0.1 parasites/μL respectively. Both of these studies targeted the rRNA genes. Recently, Farrugia *et al.*[13] described a monoplex assay that targets cytochrome *b* gene (*ctyb*) with a detection limit of 0.05 parasites/μL. Although these and other studies follow similar study methods, differences in space and time, reagents, standards used for quantification, dilution ranges, instruments or platforms, assay analysis methods, data interpretations, and much more, contribute to differences in the reported detection limits. Due to lack of consensus or standardized methods in performing qPCR, it is difficult to evaluate and/or compare the quality of work reported by different authors for a cross-study and/or cross-platform assay analysis. This also impedes the ability to reproduce reported assays.

The minimum information for publication of quantitative real-time PCR experiments (MIQE) guidelines were published recently [22]. They address the reliability of qPCR results to help ensure the integrity of the scientific literature, promote consistency between laboratories, and increase experimental transparency. The use of malaria qPCR as a confirmatory clinical endpoint assay in field research is increasing exponentially as reflected in the large number of publications reporting qPCR data. In addition, there have been discussions of molecular assays replacing microscopy as the preferred diagnostic tool for malaria, especially in reference laboratories and in CHMI trials. The lack of consensus on how to best perform qPCR has led to serious difficulty in establishing PCR as an independent yardstick [22] and currently, there is not an FDA approved qPCR assay for detection of malaria. Use of MIQE and other guidelines set-forth will ensure qPCR relevance, accuracy, correct interpretation, reliability, and reproducibility. Towards this effort, it is important to obtain harmonized data from some of currently widely used malaria qPCR assays as an important step towards assay standardization.

In this study, the performance of several published qPCR assays using the WHO international standard for *Plasmodium falciparum* DNA nucleic acid amplification technology assay as a calibration reference reagent were compared. The experimental conditions, assay analysis methods and data interpretation were uniformly performed for all qPCR assays assessed.

## Methods

### *Plasmodium falciparum* reference reagent

The WHO International Standard for *Plasmodium falciparum* DNA was used to analyse the efficiency, sensitivity and specificity of published assay targets for *Plasmodium spp*. and *P. falciparum*. The reference reagent was obtained from the National Institute for Biological Standards and Control (NIBSC; Hertfordshire, UK). This standard consists of a freeze-dried preparation of whole blood collected from a patient infected with *P. falciparum* by exchange transfusion. Following NIBSC recommendations, the lyophilized material was suspended in 500 μL of sterile, nuclease free water to a final concentration of 1 × 10,993 IU/mL, which corresponds to a parasitaemia of 9.79 parasites/100 red blood cells (RBCs) [13]. The parasite density of the WHO International Standard for *P. falciparum* DNA after the reconstitution was estimated to be 469,920 parasites/μL, based on the average RBC count of 4.8 × 106 RBC/μL. Unless otherwise indicated, fresh uninfected whole blood was used as a diluent to prepare serial dilutions. DNA was extracted using EZ1 automated purification system (Qiagen, CA, USA) using the EZ1 DNA blood kit (Qiagen, CA, USA) following the manufacturer’s recommendation. For the purposes of establishing the limit of detection (LoD), DNA was serially diluted five-fold for the first four dilution points followed by two-fold dilutions over five-log range. The lowest concentration of DNA that tested positive in all the replicates was set as the LoD.

### Primers and probes

Seven sets of primers and probes for the detection of *Plasmodium spp*. and *P. falciparum* were selected from published work. Table 1 shows the primers and probes sequences selected for this project as published. They were obtained either from Life Technologies (Carlsbad, CA, USA) or Integrated DNA Technologies (IDT-DNA, Coralville, IA, USA). For purposes of simplicity and uniformity, all probes for all the assays were labelled with 6-carboxy-fluorescein (FAM) as a reporter and 6-carboxy-tetramethylrhodamine (TAMRA) as a quencher.

**Table 1:** Published primers and probes used in analysis

**Assay name**	**Primer and Probe sets as published in 5—3 orientation**	**Ref #**
**PLU3**	**F:** GCTCTTTCTTGATTTCTTGGATG	[14]
	**R:** AGCAGGTTAAGATCTCGTTCG	
	**P:** ATGGCCGTTTTTAGTTCGTG	
**MACH**	**F**: ACATGGCTATGACGGGTAACG	[10]
	**R**: TGCCTTCCTTAGATGTGGTAGCTA	
	**P:** TCAGGCTCCCTCTCCGGAATCGA	
**CYTB**	**F:** TACTAACTTGTTATCCTCTATTCCAGTAGC	[13]
	**R:** CCTTTAACATCAAGACTTAATAGATTTGGA	
	**P:** G + TGC + TAC + CAT + GTA + AAT + GTAA	
**WHO**	**F:** CAGATGTCAGAGGTCAAATTCTAAGATT	[12]
	**R:** TCCCTTAACTTTCGTTCTTGATTAATG	
	**P:** CTGGAGACGGACTACTGCGAAAGCATTTG	
**FAL**	**F:** CTTTTGAGAGGTTTTGTTACTTTGAGTAA	[7]
	**R:** TATTCCATGCTGTAGTATTCAAACACAA	
	**P:** TGTTCATAACAGACGGGTAGTCATGATTGAGTTCA	
**PLASMO**	**F:** GTTAAGGGAGTGAAGACGATCAGA	[11]
	**R:** AACCCAAAGACTTTGATTTCTCATAA	
	**P:** ACCGTCGTAATCTTAACCATAAACTATGCCGACTAG	
**TURBO**	**F:** GTAATTGGAATGATAGGAATTTACAAGGT	[9]
	**R:** TCAACTACGAACGTTTTAACTGCAAC	
	**P:** TGCCAGCAGCCGCGGTAATTC	

Primers and probes sequences as published. Forward and reverse primers are shown as F and R. All probes shown as P were labelled with 6-carboxy-fluorescein (FAM) as a reporter and 6-carboxy-tetramethylrhodamine (TAMRA) as a quencher to maintain uniformity. The last column shows the reference where primers and probe for each assay were obtained.

### qPCR assays and experimental design

Amplification and qPCR measurements were performed using the Applied Biosystems 7500 Fast Real-Time PCR System, with version 2.0.6 software. All the analyses, including setting of the threshold and the quantification cycle (C_q_) values, were automatically established using the default settings. Experiments were performed in 96-well plates. For the TaqMan probe format, the assays were performed in the background of QuantiFast Probe Master Mix whereas in SYBR Green format, the assays were performed in the background of QuantiFast SYBR Green Master Mix and QuantiTect SYBR Green Master Mix background (Qiagen, CA, USA). The following thermal profiles described below are for each master mixes used:

#### QuantiFast Probe TaqMan

Stage 1(Holding Stage): 95°C for 5 min

Stage 2 (Cycling Stage): 95°C for 10 sec, 60°C for 30 sec} 45 Cycles

#### QuantiFast SYBR Green

Stage 1 (Holding Stage): 95°C for 5 min

Stage 2 (Cycling Stage): 95°C for 10 sec, 60°C for 30 sec} 45 Cycles

Stage 3 (Melt Curve Stage): 95°C for 15 sec, 68°C for 60 sec, 80°C for 15 sec, 60°C for 15 sec

#### QuantiTect SYBR Green

Stage 1 (Holding Stage): 95°C for 15 min

Stage 2 (Cycling Stage): 95°C for 15 sec, 60°C for 30 sec, 72°C for 30 sec } 45 Cycles

Stage 3 (Melt Curve Stage): 95°C for 15 sec, 60°C for 60 sec, 72°C for 30 sec, 60°C for 15 sec

For each set of primers and probes described in Table 1 (QuantiFast Probe TaqMan), reaction mix consisting of 11 μL of 2× QuantiFast Master Mix, 1 μL of each10 uM (0.4 μM) forward and reverse primer, and 1 μL of 5 μM probe (0.2 μM) and 6 μL of nuclease-free water was made to a total volume of 20 μL. From this, 5 μL of the mix to 1 μL template was used per reaction. For QuantiFast SYBR Green Master Mix or QuantiTect SYBR Green Master Mix, 10 μL of 2× master mix, 1 μL of each 10 μM (0.4 μM) forward and reverse primer and 8 μL of nuclease-free water was made to a total volume of 20 μL. From this, 9 μL of the mix to 1 μL template was used per reaction as recommended by the manufacturer. Good laboratory techniques and quality controls were strictly adhered. Each assay was run with proper controls in place including no template control, endogenous control and positive controls.

### Summary of published assays analysed in this study

1. Kamau *et al*[14], PLU3: Published LoD 0.0512 parasites/μL, 1 μL of DNA in 10 μL total reaction volume in 1X QuantiTect Probe RT-PCR Master Mix (Qiagen). Efficiency not reported. Performed in ABI7500 platform.

2. Lee *et al*[10], MACH: Published LoD 0.1 parasites/μL, 5 μL of DNA in 25 μL total reaction volume in 1X TaqMan universal PCR master mix (Applied Biosystems, CA, USA). Efficiency not reported. Performed in iCycler (Bio-rad) platform.

3. Farrugia *et al*[13], CYTB: Published LoD 0.05 parasites/μL, 5 μL of DNA in 20 μL total reaction volume in 1X Probe Master (Roche Molecular Biochemicals). Efficiency reported at 94.5%. Performed in a LightCycler 480 instrument.

4. Padley *et al*[7], WHO: Published LoD 16.2 parasites/μL. The amount of DNA used and reaction total reaction volume not provided however, reports amplification reactions were performed using the Light-Cycler FastStart DNA Master Hybprobe kit (Roche Applied Science, Mannheim, Germany). Efficiency not reported. Performed in a LightCycler 2.0 instrument.

5. Perandin *et al*[12], FAL: Published LoD 0.7 parasites/μL, 5 μL of DNA in 50 μL total reaction volume in 1X TaqMan universal PCR master mix (Applied Biosystems). Efficiency not reported. Performed in ABI 7700 platform.

6. Rougemont *et al*[11], PLASMO: LoD and Efficiency not published. 5 μL of DNA in 25 μL total reaction volume in 1X TaqMan universal PCR master mix (Applied Biosystems). Performed in ABI 7700 platform.

7. Hermsen *et al*[18],TURBO: Published LoD 0.02 parasites/μL, 5 μL of DNA in 25 μL total reaction volume of 25 μL of buffer containing 20 mM Tris-HCl, 100 mM KCl, 3 mM MgCl2, 0.02% gelatin, and 400 μM deoxynucleoside triphosphates (Microsynth, Balgach, Switzerland). Efficiency not reported. Performed in a GeneAMP PCR system 9700 (Applied Biosystem).

### Analysis of samples obtained from a challenge study

To further compare the assays described here, samples obtained from five subjects participating in an experimental *P. falciparum* infection study were analysed in triplicate using each of the seven assays being tested. Samples used for analysis were from positive control challenge subjects who did not receive any investigational product or licensed anti-malarial medication prior to challenge by the bite of infected mosquitoes. Samples were collected in EDTA blood and stored in -20°C immediately until needed. DNA was extracted from the whole blood using EZ1 automated purification system (Qiagen, CA, USA) with the EZ1 DNA blood kit (Qiagen, CA, USA) following the manufacturer’s recommendation. The study protocol for the clinical trial was reviewed and approved by the Human Use Review Committee of the Walter Reed Army Institute of Research (WRAIR) and by the Human Subjects Research and Review Board of the Surgeon General of the US Army at Fort Detrick, Maryland. The study was conducted in collaboration with US Agency for International Development (USAID), Infectious Disease Research Institute. Participants were provided written, informed consent before screening and enrolment and had to pass an assessment of understanding. The results of the clinical trial study will be reported elsewhere.

## Results

### Comparison of PCR assays sensitivities

**Figure 1:**
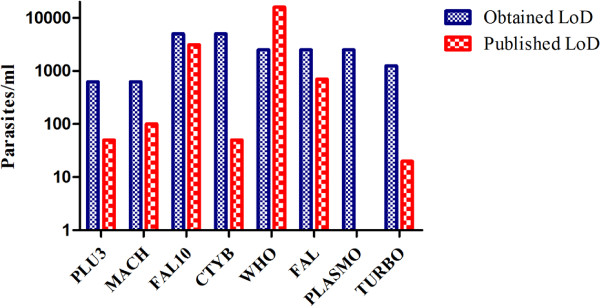
**Comparative evaluation of PCR limit of detection from published malaria PCR assays.** Data showing TaqMan probe assays performed in the background of QuantiFast Master Mix. Assays were performed in triplicate. Only C_q_ values of 40 and below were considered and only two out of three values were required to call the results a positive. LoD was established which contained the lowest parasite concentration expressed as parasites/mL.

The fluorescence emission of any molecule is dependent on environmental factors including the pH of the solution, salt concentration and much more. To obtain more accurate and representative performance of all the assays tested, assays were performed using TaqMan probe and SYBR Green formats. For the TaqMan probe format, the assays were performed in the background of QuantiFast Master Mix whereas in SYBR Green format, the assays were performed in the background of QuantiFast SYBR Green Master Mix and QuantiTect Sybr green Master Mix. All assays were performed in triplicate and the LoD was established as the highest C_q_ value where the lowest parasite concentration was detected, expressed as parasites/mL. Only C_q_ values 40 and below were considered and only two out of three assays were required to call the results a positive. It is important to note that at very low copy numbers, the normal distribution of the template in the sample is not expected. Instead, Poisson distribution is followed where only a certain percentage of copy number of the template is detected which is likely to vary each time resulting in a larger standard deviation (SD). For the TaqMan probe format, the PLU3 and MACH assays were the most sensitive with LoD of 313 parasites/mL whereas the CTYB and FAL were the least sensitive with LoD of 2,500 parasites/mL (Figure 1). The sensitivities of these assays were previously reported as follows: PLU3 at 50 parasites/mL, MACH at 100 parasites/mL, CTYB at 50 parasites/mL, WHO at 16,200 parasites/mL, FAL at 700 parasites/mL, and TURBO at 20 parasites/mL. The sensitivity of PLASMO assay was not reported.

### Comparison of the dynamic ranges and efficiencies of PCR assays

Robust and precise qPCR assays are usually correlated with high PCR efficiency [12]. To evaluate PCR efficiency, each assay was performed in triplicates, with 5-log range in five-fold decrements of the template. A slope of -3.3 ± 10% reflects an efficiency of 100% ± 10%. PCR efficiency is highly dependent on the primers used. The presence of primer dimers may result in a lower PCR efficiency in a probe-based assay and may generate false positives in SYBR Green based assays. In TaqMan probe assay format, the PLU3 assay had the highest efficiency at 100.4% whereas FAL had the lowest efficiency at 74.95% (Table 2). Efficiency for CTYB assay was previously reported as 94.5% [13] whereas in this study, efficiency of 88.2% was obtained. When the assays were run in SYBR Green format in the background of QuantiTect SYBR Green Master Mix, the PLU3 assay performed the best whereas the TURBO, WHO and CYTB assays completely failed (data not shown). Interestingly, in SYBR Green format, all assays performed better in QuantiFast SYBR Green Master Mix than they did in QuantiTect SYBR Green Master Mix. The PLU3 assay performed with the highest efficiency in SYBR Green QuantiFast Master Mix at 95.4% whereas FAL had the lowest efficiency at 40.1% (Table 3).

**Table 2:** TaqMan probe assay performance

**Assay**	**Slope**	**Y-Inter**	**R**^**2**^	**% Eff**
**PLU3**	−3.312	37.145	1	100.412
**MACH**	−3.576	38.969	0.999	90.399
**CYTB**	−3.643	37.54	0.999	88.154
**WHO**	−3.386	38.74	0.998	97.394
**FAL**	−4.116	43.601	0.998	74.954
**PLASMO**	−3.643	36.686	0.998	88.135
**TURBO**	−3.685	39.55	0.998	86.788

**Table 3:** SYBR Green QuantiFast assay performance

**Assay**	**Slope**	**Y-Inter**	**R**^**2**^	**% Eff**
**PLU3**	−3.438	35.132	0.999	95.389
**MACH**	−3.627	37.421	0.998	88.683
**CYTB**	−3.605	36.122	0.997	89.419
**WHO**	−3.585	38.815	0.999	90.095
**FAL**	−6.834	57.967	0.998	40.067
**PLASMO**	−4.07	40.183	0.998	76.088
**TURBO**	−4.092	42.658	0.998	75.551

### Comparison of R^2^ values and precision of PCR assays

Correlation co-efficient R^2^ value is a statistical term that indicates how predictive one value is of another. It is also a critical value for evaluating PCR efficiency. The value of Y (C_q_) can be used to accurately predict the value of X when the R^2^ is 1. An R^2^ value of 0.99 provides good confidence in correlating two values. Using the QuantiFast probe TaqMan assay format, the R^2^ values of all the assays were >0.99 (Table 2). There are numerous causes for variations in qPCR results, including temperature differences (temperature affects annealing and/or denaturation), pipetting errors, stochastic variation, etc. SD is a measure of precision that typically varies in qPCR with concentration and with decreasing copy number. To compare the precision of the assays tested, the SD of C_q_ values in two different dilutions containing 4,699 parasites/μL [high] and 4.7 parasites/μL [low] of the TaqMan assays were evaluated. In addition, SD at LoD was determined. The PLU3 assay had the smallest SD at all the parasite concentrations tested, which indicated superior performance whereas FAL had the poorest performance (Table 4).

**Table 4:** SD as a measure of precision of assays tested

**Assay**	**SD**	**SD**	**SD**
	**Lowest Cq values**	**Highest Cq values**	**LoD Cq values**
**PLU3**	0.044	0.249	0.000
**MACH**	0.062	0.338	0.142
**CYTB**	0.113	0.454	1.082
**WHO**	0.134	0.561	0.943
**FAL**	0.294	0.996	1.113
**PLASMO**	0.069	0.561	0.663
**TURBO**	0.096	0.509	0.200

SD is a measure of precision of each qPCR assay at different parasite concentrations. Lower values represent higher precision.

### Analysis of samples from clinical trial study

The seven assays being tested were further evaluated using clinical samples from five unvaccinated positive control subjects as described in methods section. Samples were analysed in triplicate using the TaqMan probe assay format, performed in the background of QuantiFast Probe Master Mix. Microscopy was used as the reference method for the detection of parasites and initiation of treatment whereas qPCR was used as an exploratory method. Samples were considered microscopy positive when a minimum of two unambiguous parasites were visualized and clearly identified after examining about 0.55 μL of blood in a Giemsa-stained thick smear. Table 5 summarizes the results for all assays evaluated and illustrates the day of detection for each clinical sample by each qPCR assay (first C_q_ value obtained) as compared to microscopy. The performance of each qPCR assay was assessed by comparing the number of days each assay detected parasites before microscopy. The PLU3 assay was the most sensitive, detecting parasites on average three days before microscopy, whereas the FAL assay was the least sensitive, detecting parasite only 0.6 days before microscopy.

**Table 5:** Clinical trial study

**Assay**	**ID**	**D0**	**D1**	**D2**	**D3**	**D4**	**D5**	**D6**	**DBM**	**Avg DBM**
Micro	1				P					
	2				P					
	3					P				
	4						P			
	5						P			
PLU3	1		P						2	**3**
	2		P						2	
	3		P						3	
	4		P						4	
	5		P						4	
MACH	1			P					1	**1.8**
	2		P						2	
	3			P					2	
	4				P				2	
	5				P				2	
CYTB	1			P					1	**1.6**
	2			P					1	
	3			P					2	
	4				P				2	
	5				P				2	
WHO	1		P						2	**2**
	2			P					1	
	3		P						2	
	4				P				2	
	5			P					3	
FAL	1				P				0	**0.6**
	2				P				0	
	3				P				1	
	4					P			1	
	5					P			1	
PLASMO	1			P					1	**1.2**
	2			P					1	
	3				P				1	
	4					P			1	
	5				P				2	
TURBO	1		P						2	**1.6**
	2			P					1	
	3				P				1	
	4				P				2	
	5				P				2	

Table showing the number of Days (D) the samples became positive (P) by either microscopy (Micro) or qPCR assays. D1 is the day when the first qPCR assay was positive. The designation of days is arbitrary for comparison of qPCR assays to microscopy and does not reflect the actual day of qPCR positivity post infection challenge. DBM is the number of days samples were qPCR positive before they became microscopy positive. The actual C_q_ values are not shown. The last column shows the average number of days (Avg DBM) the samples were qPCR positive (for each assay) before they were microscopy positive for all the five volunteers.

## Discussion

A variety of diagnostic methods exist that are used for identification and speciation of *Plasmodium* parasites. Microscopic detection of malaria parasites on Giemsa-stained blood smears is still considered the gold standard method for malaria diagnosis, clinical trials efficacy evaluation and epidemiological surveys. However, microscopy has numerous limitations such as low sensitivity, difficulties in quality control and standardization, operator dependence, poor specificity, and the need for continued training and evaluation [4,6,23,24]. Identification of parasites specific antigens, antibodies and nucleic acid sequences form the basis of established and novel diagnostic modalities. Nucleic acid amplification technique-based assays, such as qPCR are becoming increasingly employed in the diagnosis of malaria [12,15,25,26]. Studies have clearly demonstrated that qPCR methods have improved sensitivity and species identification compared to microscopy [7,14]. There is a wide variation in the sensitivity of the numerous PCR methods that have been developed for the laboratory diagnosis and clinical management of malaria [21]. These differences may be attributed to the intrinsic variability in assay sensitivity or a consequence of calibration using different reference reagents, which are poorly standardized [12]. In this study, the WHO International Standard for *P. falciparum* DNA was used as a calibration reference reagent to compare the sensitivity of a few published qPCR assays for detection of malaria. The MIQE guidelines were followed during experiment set-up and execution to ensure relevance, accuracy, correct interpretation, and repeatability of the assays that were being analysed and compared. The PLU3 assay performed extremely well and was consistent compared to the other assays regardless of the assay format (either TaqMan probe or SYBR green) or background (QuantiFast probe Master Mix, QuantiFast SYBR green Master Mix or QuantiTect SYBR green Master Mix). The MACH assay performed equally well. There is probably other qPCR malaria assays not included, published or not, that might perform as well or better. With exception of the WHO assay, all the qPCR assays tested had higher LoD (less sensitive) compared to the published LoD. This difference can be explained by many factors including calibration using different reference reagents and data interpretation.

Although sensitivity is important, other factors should be considered when designing or selecting qPCR assay to adopt or use in a project. *Plasmodium falciparum* growth is usually characterized by an exponential increase in the number of parasite-infected erythrocytes, followed by marked oscillations in this number with a periodicity of 48 hours, which are eventually dampened [27]. This reproductive pattern leads to variation in parasite densities in peripheral blood. As such, the small differences in assay sensitivity demonstrated here might not be clinically relevant. In addition, different settings may require qPCR assays with different sensitivities. The severity of malaria disease does not just depend on the parasite density, but depends on many factors such the immune status of the patient or subject in addition to other factors. For non-immune patients or subjects, smaller amounts (or changes thereof) of parasites may cause malaria disease much more compared to immune patients or subjects, therefore requiring qPCR with higher sensitivity. However, regardless of the setting, qPCR assays must be consistent and reproducible in addition to being highly sensitive; these qualities can be established if qPCR assays are designed and tested following the MIQE guidelines.

Some of the key characteristics that are critical in qPCR experiments and must be considered conceptually include analytical sensitivity, analytical specificity, accuracy, repeatability, and reproducibility. It is important that published assays are reproducible within reason or range if performed following the same chemistries and platform as those described in respective publication. Even with change in chemistries and platform, the performance of the assay should remain relatively consistent. Such characteristic (consistency) in performance can only be evaluated if the assay calibration is done using a standard reference reagent, such as the WHO International Standard for *P. falciparum* DNA. The quality and integrity of the nucleic acids being analysed is just as critical in performance of the assay. Therefore, use of standard controls, such as the WHO International Standard for *P. falciparum* DNA, in every qPCR assay as positive control is critical in ensuring that the qPCR assays perform consistently. To ensure consistency and minimize variability, all the assays tested here were performed on the same 96-well plate when possible and the same DNA dilutions were used in all the assays.

Data analysis is also important in ensuring impartial results. In most qPCR platforms, the post-run data are analysed using the software supplied with the instrument. Proper baseline and threshold setting is required in getting a final quantifiable C_q_ value for each run. Such settings can be done manually or automatically in open qPCR platforms. Manually changing the threshold settings can drastically change the C_q_ values. It is likely that such practices account for dramatic differences seen in obtained and published LoD. To ensure that there was no bias towards data interpretation and analysis in this study, automatic manufacturer’s settings were used to establish threshold and for data interpretation.

The performance of the assays in analysis of the clinical trial samples corresponded well with the established base-line performance of the assays. The PLU3 assay which had been established to be the most sensitive, detected parasites on average three days before microscopy followed by the MACH assay. The FAL assay was the least sensitive; had the lowest performance in the analysis of clinical samples and had the worst LoD. These data show that proper evaluation of molecular assays following proper guidelines results in an assay with reliable, reproducible and superior performance.

## Conclusion

For the first time, the performances of several PCR assays developed by different laboratories for detection of malaria have been compared side by side. To ensure unbiased and objective comparison of the performance of the qPCR assays, data was generated with clearly defined experimental design, procedures and instrumentation for DNA extraction, as well as the analysis. In addition, qPCR assays tested were validated using MIQE guidelines and commercially available reference DNA sample. When designing and evaluating qPCR assays, the focus should be the chemistries regardless of the background and platform used. Data presented here show that qPCR assays with superior performance characteristics such as high efficiency and precision perform better in analysis of clinical samples than those with poor performance characteristics. With exception of the WHO assay, qPCR assays analysed did not perform with similar sensitivities as previously shown. It is recommended that the work described here, either using the same and/or additional malaria qPCR assays be performed by other group(s) as well. It is absolutely important that such testing and comparison of the performances of qPCR assays use well defined guidelines such as MIQE and same reference reagent(s) such as the WHO International Standard for *P. falciparum* DNA. Reference reagent(s) can also be prepared internally but the same reagent(s) must be shared and used by all the participating laboratories in such a study.

The purpose of this study was not to endorse or discredit any of the published assays. It is likely most established laboratories will continue using their laboratory developed qPCR assays for detection of malaria. However, it is critical to reach a consensus or standardized method of performing qPCR assay to facilitate the evaluation and/or comparison of the qPCR assays reported by different authors and laboratories. This will be especially important for a cross-study and/or cross-platform comparison.

## Competing interests

The authors declare that they have no competing interests.

## Authors’ contributions

EK, SA and CFO conceived and designed the experiments. SA, KCF and EK performed the experiments. SA and EK analysed the data. JC, JK and CFO contributed reagents/materials/analysis tools. EK and SA wrote the Manuscript: EK, SA. KCF, JK, JC, and CFO reviewed the manuscript. All authors read and approved the final manuscript.
